# The divergence of mean phenotypes under persistent Gaussian selection

**DOI:** 10.1093/genetics/iyaf031

**Published:** 2025-02-25

**Authors:** Michael Lynch, Scott Menor

**Affiliations:** Biodesign Center for Mechanisms of Evolution, Arizona State University, Tempe, AZ 85287, USA; Biodesign Center for Mechanisms of Evolution, Arizona State University, Tempe, AZ 85287, USA

**Keywords:** evolutionary divergence, Gaussian selection, mutation bias, phenotypic divergence, phenotypic scaling, random genetic drift, selective interference, stabilizing selection

## Abstract

Although multigenic traits are often assumed to be under some form of stabilizing selection, numerous aspects of the population-genetic environment can cause mean phenotypes to deviate from presumed optima, often in ways that effectively transform the fitness landscape to one of directional selection. Focusing on an asexual population, we consider the ways in which such deviations scale with the relative power of selection and genetic drift, the number of linked genomic sites, the magnitude of mutation bias, and the location of optima with respect to possible genotypic space. Even in the absence of mutation bias, mutation will influence evolved mean phenotypes unless the optimum happens to coincide exactly with the mean expected under neutrality. In the case of directional mutation bias and large numbers of selected sites, effective population sizes ($N_{e}$) can be dramatically reduced by selective interference effects, leading to further mismatches between phenotypic means and optima. Situations in which the optimum is outside or near the limits of possible genotypic space (e.g. a half-Gaussian fitness function) can lead to particularly pronounced gradients of phenotypic means with respect to $N_{e}$, but such gradients can also occur when optima are well within the bounds of attainable phenotypes. These results help clarify the degree to which mean phenotypes can vary among populations experiencing identical mutation and selection pressures but differing in $N_{e}$, and yield insight into how the expected scaling relationships depend on the underlying features of the genetic system.

## Introduction

Even under persistent long-term forms of stabilizing or directional selection, the mean phenotypes of independent lineages are expected to diverge to a degree that depends on the distribution of fitness effects of mutations, the genetic architecture of the trait, the level of mutation bias, and the genetic effective population size ($N_{e}$), which itself can depend on all of the above. Provided classes of mutations exist that are accessible to selection in some lineages but effectively invisible in others (owing purely to insufficient effective population size), gradients in phenotypic features are then expected among populations with respect to $N_{e}$, even in the case of invariant selection. These issues have been taken up in detail for the special case of multiplicative fitness functions, wherein each mutation influences fitness independently of all others, i.e. there are no epistatic fitness effects (Lynch 2018; Devi *et al.* 2023), and to a lesser extent for traits under alternative forms of selection (Charlesworth 2013; Lynch 2020).

Nevertheless, it is commonly believed (although still debated; Schluter 1988; Kingsolver and Diamond 2011; Urban *et al*. 2013) that complex quantitative traits are typically under a form of stabilizing selection operating around an optimal intermediate phenotype. Most often, the fitness function is assumed to be Gaussian (bell-shaped) in form, if for no other reason than to simplify mathematical analysis (Walsh and Lynch 2018). Fitness functions with stabilizing features introduce several novel issues with respect to understanding the positions of drift barriers, i.e. the limits to which mean phenotypes are expected to disperse under the joint influences of selection, drift, and mutation.

Under Gaussian selection, the fitness gradient is a function of the location of the mean phenotype relative to the optimum. If the mean phenotype coincides with the optimum, then mutations with increasingly large phenotypic effects will have negative fitness effects that accelerate in a nonlinear fashion. However, from the standpoint of individual genotypes, the fitness effects of mutations are context-dependent, with mutations with sufficiently small effects oriented in the right direction improving fitness, but others having deleterious effects (Fisher 1930; Orr 2006). In this sense, stabilizing fitness functions introduce epistasis, in that the selective effects of mutations depend on the genetic backgrounds of their carriers (even if the underlying phenotypic effects of mutations are additive). These issues become increasingly important if the mean phenotype deviates from the optimum, as is expected whenever the latter does not coincide with the neutral expectation.

Here, we examine the responses of drift barriers to changes in $N_{e}$ for traits experiencing persistent selection under a Gaussian fitness function. The goal is to evaluate whether perceptible differences in mean phenotypes are expected to arise among species solely as a consequence of changes in the power of random genetic drift, in the absence of changes in underlying selection pressures. In its most general form, this treatment yields insight into a number of different selection scenarios. If the optimum phenotype is intermediate to the range of phenotypes that can be attained by mutation, and there is no mutation bias, mean phenotypes drift around the optimum to a degree that depends on $N_{e}$, with the grand mean coinciding with the optimum, i.e. selection is ultimately stabilizing in nature. If, on the other hand, the optimum coincides with the most extreme phenotype that can be produced by mutation, then selection is always directional in nature, albeit with diminishing strength as the mean phenotype approaches the extreme. In this case, a $N_{e}$-dependent gradient in mean phenotypes is expected to arise, to a degree that depends on the degree of linkage, the distribution of strength of selection operating on individual genomic sites, and the magnitude of mutation bias. More generally, even with an intermediate optimum phenotype, mutation pressure will often result in a discordance between the optimum and evolved mean phenotypes, and this results in selection operating in a predominantly directional manner, as the steady-state phenotype distribution is largely nonoverlapping with the optimum.

Our work is related to prior efforts to derive expressions for the load on mean population fitness resulting from the joint operation of mutation, selection, and random genetic drift (e.g. Barton and Coe 2009; Roze and Blanckaert 2014; Barton 2017; Vanhoenacker *et al*. 2018). Although this approach has a long history in evolutionary genetics, the predicted load on fitness is typically so low that it would be undetectable in empirical studies, which would also be confronted with the problem of how to measure fitness. Our goal is to determine when, on the phenotypic scale, there is a sufficiently broad range of variation of mean phenotypes to be detectable with comparative data and useful for downstream hypothesis testing (for example, evaluating whether mean phenotypes scale with respect to effective population sizes).

### The model

Throughout, we deploy computer simulations and develop mathematical approximations to develop insight into the ways in which the strength of selection, mutation rates and bias, size of linkage groups, and effective population size influence the long-term steady-state behavior of the distribution of mean phenotypes under constant environmental conditions. The basic structure of the model is similar to that utilized in prior studies with an exponential fitness function (Lynch 2020; Devi *et al*. 2023), except that here we focus on a Gaussian fitness function, where the fitness effects of mutations depend on the distance of the recipient phenotype from the optimum. This contrasts with an exponential fitness function, wherein the effects of mutations are independent of the genetic background.

As a surrogate for understanding some effects of recombination, we consider single linkage blocks of *L* nonrecombining sites (or genetic loci), each with biallelic states, + and −, contributing positively and negatively to the trait in an additive fashion, although the magnitude of +/− effects is allowed to vary among sites in some applications. Because the stretch of nucleotide sites under consideration is assumed to be completely linked, the positions of the sites are irrelevant, and there can be a multiplicity of functionally equivalent haplotypes (i.e. with identical numbers of + alleles) in each effect class, which modifies their ease of mutational accessibility (Lynch 2018, 2020). For example, there are just single haplotype configurations with all + or all − alleles, but *L* haplotypes with a single + or single − alleles, L(L−1)/2 haplotypes with two + and (L−2) − alleles (and vice versa), etc. The site-specific per generation mutation rates from the − to the + states, and vice versa, denoted as $u_{01}$ and $u_{10},$ respectively, are assumed to be identical at all sites. We refer to genomic sites in a generic sense, and these may be viewed as either individual nucleotide positions or genetic loci provided they are effectively biallelic at the phenotypic level (e.g. at the DNA level, this could include situations in which three of the four nucleotides have the same phenotypic effects, or two pairs are internally equivalent).

Given the assumptions of additive mutational effects and complete linkage, the following analyses do not encompass the full range of possible genetic architectures of traits. They should, however, apply to a wide array of situations, including the genome-wide distributions of sites underlying traits with a largely additive genetic basis in asexual species or those that only rarely engage in sexual reproduction. For situations in which recombination is more frequent, the results should still apply provided the size of the linkage block is small enough to avoid recombination over the duration of a typical genealogical coalescence (on the order of $N_{e}$ generations; Good *et al*. 2014; Devi *et al*. 2023). In the following analyses, we draw comparisons from prior work that assumes the opposite extreme of free recombination between all sites. (We have not evaluated the consequences of multiple, independent linkage blocks within genomes.)

One concern is that mutations within genes are more likely to have epistatic (nonadditive) effects with each other than between-gene interactions, but incorporation of such effects into the analyses would introduce multiple layers of complexity, owing to the alternative forms of epistasis. Such exploration is desirable, but is beyond the scope of this study, and we refer to Barton (2017) for some reasons why such effects are unlikely to alter our qualitative conclusions, e.g. in the absence of recombination, mean population fitness is independent of the level of epistasis (Kimura and Maruyama 1966), which implies the evolution of a constant distance of the mean phenotype from the optimum regardless of the level of epistasis.

To perform analyses with biologically realistic combinations of parameter values, we largely adhere to the scaling relationship of mutation rates and coalescent effective population sizes known to exist across the Tree of Life. The latter generally fall in the range of $10^{4}$ to $10^{9}$, and the mutation rate per nucleotide site scales negatively with the ∼0.76 power of *N*, where the latter denotes the coalescent effective population size determined from standing levels of heterozygosity at silent sites in protein-coding genes (Lynch *et al*. 2016, 2023). Thus, where computational work was involved, the following analyses were performed under the assumption of a per-site mutation rate from + to − alleles of $10^{-7}$ at a population size of $N=10^{4}$, with $u_{10}=0.0011\;N^{-0.76}$. With this scaling, for the full range of population sizes employed here ($N≃10^{4}$ to $2\times 10^{8}$), the product $Nu_{10}$ then ranges from ∼0.01 mutations/population/site/generation at the lowest to ∼0.10 at the highest population sizes, consistent with the weak scaling of population-level silent-site diversity for diverse organisms (Walsh and Lynch 2018). Nonetheless, using the analytical approximations that we develop below, one can still directly explore the consequences of arbitrary combinations of mutation rates and population sizes.

We evaluate the behavior of linkage block lengths ranging from 10 to $10^{4}$ and mutational biases $\beta =u_{01}/u_{10}=0.10$ to 1.00. This definition of mutation bias is a natural one in that it is a function of allelic states. At the level of haplotypes, there can still be a realized mutation bias even if β=1, but this is totally a function of differences in numbers of + vs. − alleles in the prevailing haplotypes. Naturally, the range of variation of realized mutational effects depends on the haplotype distribution, as the directionality of overall effects depends on the extent to which the population is saturated by + vs. − alleles.

The trait under consideration is assumed to have an additive genetic basis at the phenotypic level, so that in situations in which all alleles are scaled to have effects of 0 and 1 (as done here), the genotypic value of a haplotype with $n_{+}$ plus alleles is


(1) $$z=n_{+}=L-n_{-}.$$

Under the Gaussian selection model, the fitness of an individual with genotypic value *z* is


(2) $$W(z)=\exp\;[-s(z-\theta_{s}{)}^{2}],$$

where $\theta_{s}$ is the optimal genotypic value imposed by the selective environment, and *s* is a measure of the strength of selection. (In principle, the phenotypic value need not equal the genotypic expectation, but this matter is accommodated by noting that environmental sources of noise are absorbed into the selection coefficient *s*, which diminishes with decreasing correspondence between genotype and phenotype.) Under this model, fitness declines monotonically with increasing distance of the genotypic value from the optimum. Although the selective differences between adjacent genotypes are nonconstant, the reduction in fitness between the optimal genotype and neighbors deviating by single phenotypic units, i.e. $|z-\theta_{s}|=1$, is ≃s for s≪1 (Appendix A). Note that our *s* is equivalent to $1/(2V_{s})$ used in numerous other quantitative-genetic expressions in the literature. Throughout, we examine the consequences of a range of selection strengths from $s=10^{-6}$ to $10^{-2}$.

We rely on a classical Wright–Fisher discrete-generation model with sequential episodes of mutation, selection, and random genetic drift, with a constant population size of *N* haploid individuals, so that all new mutations have initial frequencies of 1/N. Assuming additive effects on phenotypes, all results should extend to diploids by substituting 2N for *N* in the following expressions. As will be discussed below, the actual genetic effective size of the population ($N_{e}$) can be influenced further by the structure of the linkage group, the strength of selection, and *N* itself, as these factors influence the degree of selective interference among simultaneously segregating mutations (Devi *et al*. 2023).

Because mutations are reversible, this system always eventually evolves to a quasi-steady-state distribution of mean phenotypes, provided the fitness function remains constant. Owing to the stochastic nature of the underlying processes, to obtain stable estimates of the steady-state distributions, computer simulations must proceed for very large numbers of generations. Thus, to achieve greater computational speed, for large population sizes, we often scaled the input parameters so as to keep $Nu_{10}$, $Nu_{01}$, and *Ns* constant, by reducing *N* and increasing the mutation and selection parameters by the same factor, with constraints such that *N* was always $\geq 10^{3}$, and *s* and $Lu_{10}$ always ≤0.1. Burn-in periods before compiling statistics were typically at least $10^{3}N$ generations, with the haplotype constitutions of populations being assayed every N/10 generations thereafter, typically for $10^{6}$ to $10^{8}$ intervals.

Simulations were carried out with a program written in C++, in a form that allows parallel analysis of multiple population sizes, using the same general procedures as in Devi *et al*. (2023), and freely available at https://github.com/LynchLab/Asexual-Gaussian-Selection. Given the focus on linkage blocks, the frequencies of the L+1 haplotype classes were monitored through time in discrete generations. Mutations to adjacent classes were assigned based on the numbers of + and − alleles and the respective mutation rates, and this was then followed by selection defined by the Gaussian fitness function weighted by mean population fitness. Random genetic drift was then imposed by multinomial sampling across the surviving haplotype distribution, returning the population to the next round of mutation, selection, and drift.

We note that beyond our own prior work, previous studies have focused on a biallelic genetic system like that employed here (e.g. Barton 1989; Walsh and Lynch 2018; and the many references therein). For example, as reviewed and expanded upon by Roze and Blanckaert (2014), Barton (2017), and Vanhoenacker *et al*. (2018), considerable attention has been given to the reduction in mean population fitness resulting under various scenarios involving stabilizing selection, often with an optimum intermediate to the range of possible phenotypic variation, and under the assumption of free recombination and no mutation bias. Although related, the focus here is on the effects of linkage and mutation bias on the scale of mean phenotypes. As described below, we will make use of one particularly useful application of a Gaussian fitness function with mutation bias by Charlesworth (2013), who assumed free recombination.

## Results

In the following sections, we progressively evaluate scenarios of increasing (and biologically realistic) complexity, starting with the situation in which all sites have equivalent effects and the optimum phenotype is well embedded within the possible range of variation, a common scenario invoked in previous work. We then consider situations in which the optimum phenotype is moved closer to the edge of possible phenotypic variation. Finally, we illustrate the consequences of sites with variable effects on the trait.

### Homogeneous sites, with an intermediate optimal phenotype

The most commonly employed model in evolutionary quantitative genetics relies upon a Gaussian fitness function with an optimum assumed to fall well within the range of phenotypic variation (Walsh and Lynch 2018). There can, however, be some subtleties even with this simple scenario (Barton 1986, 1989). In particular, for a model in which mutations have discrete effects, the optimum may not coincide exactly with any possible summation of allelic effects, and there may be multiple equilibria. The most extreme case arises when a single site determines the trait value, with the optimum residing at the midpoint between the effects of the two alleles, which renders both alleles neutral with respect to each other. Less extreme effects of this nature occur with increasing numbers of underlying sites. In the following, we focus on situations in which the optimum is attainable, with *L* being an even number (initially with $\theta_{S}=L/2$) and the effects of − and + alleles being 0 and 1, respectively.

*No mutation bias (β=1).* Under this model, if mutations have no directional bias, i.e. $u_{01}=u_{10}$, the grand mean phenotype is expected to evolve to the optimum, as this is also the position of mutation equilibrium. The focus is then on the mean absolute deviation from the optimum, as the temporal means wander above and below the optimum in equal frequencies. In the following, the average absolute deviation of phenotypic means from the optimum is denoted as $\bar{\left|\delta \right|}$, which is equivalent to $\sqrt{2/\pi }$ times the standard deviation of the distribution of phenotypic means if the latter is Gaussian in form.

**Fig. 1.**
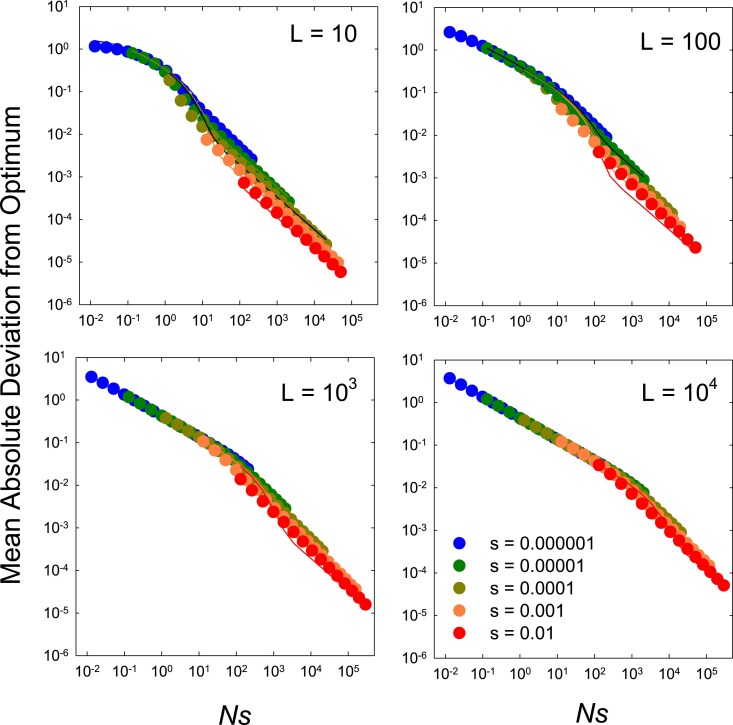
Average absolute deviation of phenotypic means from the optimum, $\bar{\left|\delta \right|}$, for the case in which the optimum is located in the middle of the range of possible variation, $\theta_{S}=L/2$, and there is no mutational bias, β=1.0. Points were obtained by computer simulations. Lines are predictions from Equations (6)–(8), and for the most part, they are hidden by the dots, as the fits are excellent. For Ns<L/20, the mean deviation is simply a function of *NLs*, as given by Equation (6), whereas for larger *Ns*, it converges on values that depend only on $u\sqrt{L}/s$, as defined by Equation (7).

With unbiased mutation and population means evenly distributed around $\theta_{S}$, there is no expected gradient in the overall mean phenotype with increasing *Ns*. Not surprisingly, however, the mean absolute deviation declines rapidly with an increase in the composite parameter *Ns*, which is equivalent to the ratio of the strength of selection to the power of drift (assuming $N_{e}=N$) (Fig. 1). It also increases with the size of the linkage block, although not greatly so, as explained below. There is an upper bound to the mean deviation from the optimum at Ns≪1, as populations then converge on the neutral expectation for the dispersion of mean phenotypes, defined by the balance between forward and reverse mutations, although the effect is only noticeable at low *L*.

To explain these results in a more mechanistic way, note that a diffusion approximation applied to a quantitative-genetic model suggests that the distribution of mean phenotypes should be proportional to the product of the expectation under neutrality and the fitness of each genotypic class taken to the 2N power (Lande 1976; Lynch 2018; Appendix B). The distribution is expected to be approximately Gaussian in form with expected means and variances of phenotypic means equal to


(3) $$\mu (\bar{z})=\frac{\kappa \theta_{S}+\theta_{N}}{\kappa +1},$$

(4) $$\sigma^{2}(\bar{z})=\frac{\sigma_{N}^{2}}{\kappa +1},$$

where $\theta_{N}=L\beta /(1+\beta )$ is the expected mean phenotype under selective neutrality, and


(5) $$\kappa =\frac{\sigma_{N}^{2}}{\sigma_{S}^{2}}$$

is the ratio of the expected variance of the distribution of means under mutation pressure alone vs. under conditions in which selection is the prevailing force (both of which are further described in terms of the underlying population-genetic parameters in Appendix B). For in the case of no mutational bias, κ≃2NLs, the grand mean phenotype coincides with $\theta_{S}$, and the mean absolute deviation simplifies to


(6) $${\bar{\left|\delta \right|}}_{1}≃\sqrt{\frac{L}{\pi (2NLs+1)}}$$

(Appendix C). This suggests that the mean absolute deviation from the optimum is a function of the ratio of the strengths of selection and drift (*Ns*) and of the number of selected sites (*L*), independent of the mutation rate. With sufficiently large numbers of sites (NLs≫1), Equation (6) further reduces to $(2\pi Ns{)}^{-0.5}$, so that the magnitude of drift from the optimum is expected to be inversely related to the square root of *Ns*, superficially appearing to be independent of *L*.

A potential issue here is the need to distinguish between effective and absolute population sizes, $N_{e}$ vs. *N*, as interference between simultaneously segregating mutations is expected to reduce the efficiency of selection relative to the case of free recombination. For the question at hand, $N_{e}$ can be defined as the population size required for Equation (3) to yield an estimate of the mean phenotype equivalent to that obtained by computer simulations. Letting $N_{e}=\phi N$, and substituting the latter for *N* in the expression for *κ*, one can then solve for the value of *ϕ* that yields a match between the observed and predicted mean deviation. For the current case of β=1, we find that over the full range of parameter space evaluated in Fig. 1, $N_{e}$ is never depressed more than 15% below *N*, and for most cases is essentially equal to the latter, i.e. 0.85<ϕ≤1.00. (By embedding a neutral site within haplotypes during simulations, we also found that the neutral heterozygosity was depressed by no more than 15%). Thus, for the special case of no mutation bias around an intermediate optimum phenotype, there is minimal selective interference, and Equation (6) provides a very good fit to the simulation data using $N_{e}=N$, although only up to Ns≃L/20.

For *Ns* larger than this critical value, the population behaves in a near deterministic fashion with most sites in selection–mutation balance, with those marginally above and below the optimum wandering very slightly in time (Barton 1989). The absolute mean deviation then becomes a matter of the average difference in the number of sites segregating for deleterious mutations above and below the optimum, which we find to be


(7) $${\bar{\left|\delta \right|}}_{2}≃\frac{2u\sqrt{L}}{s}$$

for Ns≫1. A general expression allowing a transition between these two domains of behavior,


(8) $$\bar{\left|\delta \right|}={\bar{\left|\delta \right|}}_{2}+({\bar{\left|\delta \right|}}_{1}-{\bar{\left|\delta \right|}}_{2})e^{-2Ns/L},$$

provides an excellent fit to the simulated data over the full range of *Ns* (Fig. 1). (Note that $\bar{\left|\delta \right|}$ declines with increasing *Ns* in Fig. 1 simply because the mutation rates deployed in the simulations are scaled to decline with increasing *N*.)

*Effects of mutation bias.* Mutation bias introduces complications in the preceding theory because the steady-state distribution of mean phenotypes is no longer symmetrical around the optimum and may even be essentially nonoverlapping. Provided the distribution of means remains approximately Gaussian, Equations (3) and (4) (further developed in Appendices B and D) can still be used to estimate $\bar{\left|\delta \right|}$, although the need to account for the fact that $N_{e}<N$ becomes more significant.

Under the assumption that mutation bias is strong enough that $\theta_{s}$ falls outside of essentially the entire range of variation of mean phenotypes, Charlesworth (2013, his Equation 9) derived an expression for the expected deviation of the grand mean from the optimum for the special case of free recombination and NLs≫1 (satisfied in almost all of our analyses),


(9) $$\delta ≃\frac{1-\ln\;(\beta )}{4Ns}.$$

This provides a benchmark for considering the effects of linkage, as implemented here.

**Fig. 2.**
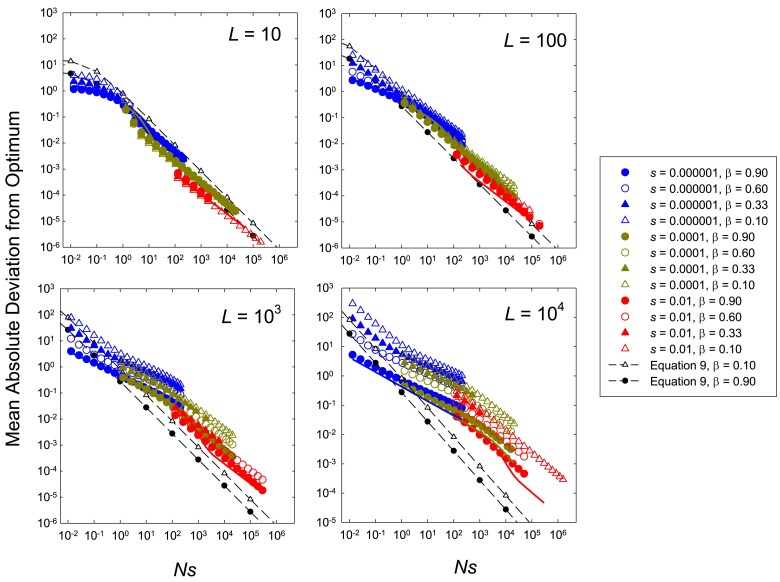
Results analogous to the plots in Fig. 1, but for situations involving four levels of mutation bias (β<1.0). The dashed lines are the expectations from Equation (9) (which assumes free recombination, and a strong deviation of the overall phenotypic mean from the optimum), given for low and high mutation biases (β=0.9, lower black dots, and 0.1, upper white triangles). The thick solid lines (where visible) are the analytical expectations, using Equations (6)–(8), which assume no mutation bias (β=1.0).

Simulation results indicate that *Ns* is still a primary factor in determining the absolute mean deviation from the optimum, and that $\bar{\left|\delta \right|}$ is progressively increased with increasing degrees of mutational bias, especially when linkage blocks are large ($L=10^{3}$ and $10^{4};$Fig. 2). However, there is an additional effect of *s* beyond that coming through *Ns*. For small numbers of sites (L=10), Equations (6)–(8) (which assume β=1) continue to provide adequate fits in all cases unless population sizes are extremely small (Ns≪1) and mutation is extremely biased (in which case, the distribution gravitates towards the neutral expectation). Equations (6)–(8) also provide excellent fits to the data for all cases when mutation bias is weak (β=0.9), but can yield large underestimates of $\bar{\left|\delta \right|}$ as *β* declines further. Although Equation (9) implies scaling behavior of $\bar{\left|\delta \right|}$ with *Ns* that is qualitatively consistent with the simulation results when L<10, it becomes increasingly problematic with larger linkage blocks, where the decline in actual $\bar{\left|\delta \right|}$ with *Ns* becomes shallower and the observed deviations become increasingly underestimated (by one to two orders of magnitude).

**Fig. 3.**
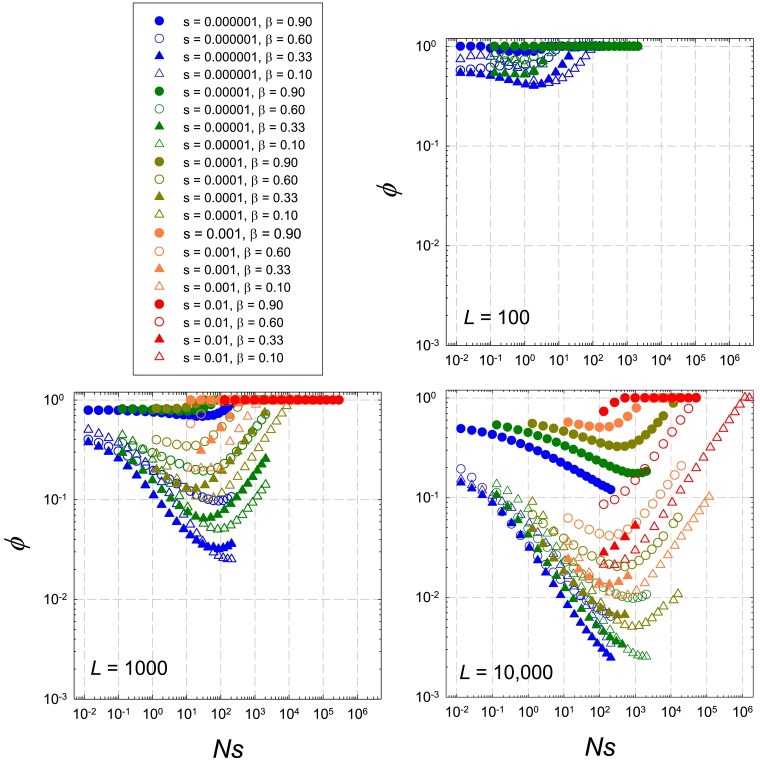
Estimates of $\phi =N_{e}/N$ obtained from the application of computer-simulation data to Equation (3), as a function of *Ns*, *L*, *β*, and *s* for the case in which the optimum $\theta_{S}=L/2$ is intermediate on the scale of *L*.

A more revealing understanding of the behavior of $\bar{\left|\delta \right|}$ can be achieved by considering the ways in which the effective population size is influenced by the underlying processes. As can be seen in Fig. 3, for L<100, observed $\phi =N_{e}/N$ [obtained from the simulated data, by optimizing the fit to Equation (3)] is nearly always larger than 0.5, but for larger linkage blocks, there is a pronounced u-shaped response of *ϕ* to increasing *Ns*. *ϕ* converges to 1.0 at very low *Ns* because selection is no longer effective, and also converges to 1.0 at very high *Ns* because selection is so effective that there are few segregating polymorphisms to cause selective interference. For 1<Ns<100, *ϕ* can decline below 0.1 and even approach $10^{-3}$.

Semi-analytical expressions for *ϕ* in terms of the model parameters, developed in Appendix E using approaches previously introduced in Devi *et al*. (2023), take into consideration the numbers of parallel, competing mutations arising during the expansion of a potentially favorable mutation. These expressions generally lead to estimates of *ϕ* that are within a factor of two of observed estimates (Supplementary Fig. 1), provided Ns<L/20 (the deterministic limit), and therefore yield insight into the general underlying determinants of the selective interference reducing the effectiveness of stabilizing selection. For very strong and effective selection, Ns≫L/20, a generalization of Equation (7) with (1+β) substituted for 2 provides a good estimate of the mean deviation. Thus, Equations (3), (6), and (8) work well upon substitution of an appropriately defined measure of $N_{e}$ for *N*.

Although somewhat complicated algebraically, the formulae for *ϕ* [Equations (E6a)–(E7b)] indicate that when the phenotypic optimum is intermediate on the scale of *L*, *ϕ* declines in a nonlinear way with increasing values of the composite bias parameter $\beta (1-\beta )/(1+\beta {)}^{2}$. Thus, the effect of mutational bias is nonmonotonic in *β*, reaching a minimum when β=0.33, consistent with the observations for *ϕ* in Fig. 3. For large linkage blocks and relatively weak selection (Ns≪L/20), *ϕ* also decreases with increasing strength of the population-level mutation rate ($LNu_{10}$) and with the ratio of the strength of selection relative to drift (*Ns*). In this domain, a separate effect of *L* also enters through the steady-state distance of the grand mean from the optimum, yielding an approximate overall scaling of *ϕ* with $[(LN{)}^{2}u_{10}s{]}^{-1/3}$ [Equation (E7b)].

Finally, it is also notable that the behavior of *ϕ*, which relates to fixation probabilities, is not reflected in levels of depression of standing variation at linked neutral sites (which we determined by embedding a neutral marker into the haplotypes), consistent with earlier findings with an exponential fitness function (Devi *et al*. 2023). That is, the $N_{e}$ governing divergence is not the same as the coalescent effective population size that is conventionally inferred from measures of silent-site variation in natural populations; for the latter, *ϕ* rarely drops below 0.25 in any of the preceding analyses.

### Nonintermediate optimum

Although it is common practice among theoreticians to model Gaussian selection as though the optimum (and the range around it) resides well within the range of possible phenotypic variation, there is no reason to think that cases of asymmetry (with or without the optimum within the bounds of the possible genotypic range) are uncommon. Such conditions can greatly alter the degree to which mean phenotypes can drift from the optimum. Consider, for example, the case of a half-Gaussian fitness function, such that the optimum genotypic state contains a + allele at each site, i.e. $\theta_{S}=L$. In this situation, selection operates in a purely directional manner, with all − alleles being unconditionally deleterious. The mean phenotype will then always be $\leq \theta_{S}$, so that the mean deviation below the optimum is also the average absolute deviation. Thinking more generally, so that $\theta_{S}=xL$, as *x* declines from 1 to 1/2 (the case of an intermediate optimum), the mean phenotype will be increasingly prone to drifting both above and below the optimum, to a degree dictated by the strength and direction of mutation bias.

**Fig. 4.**
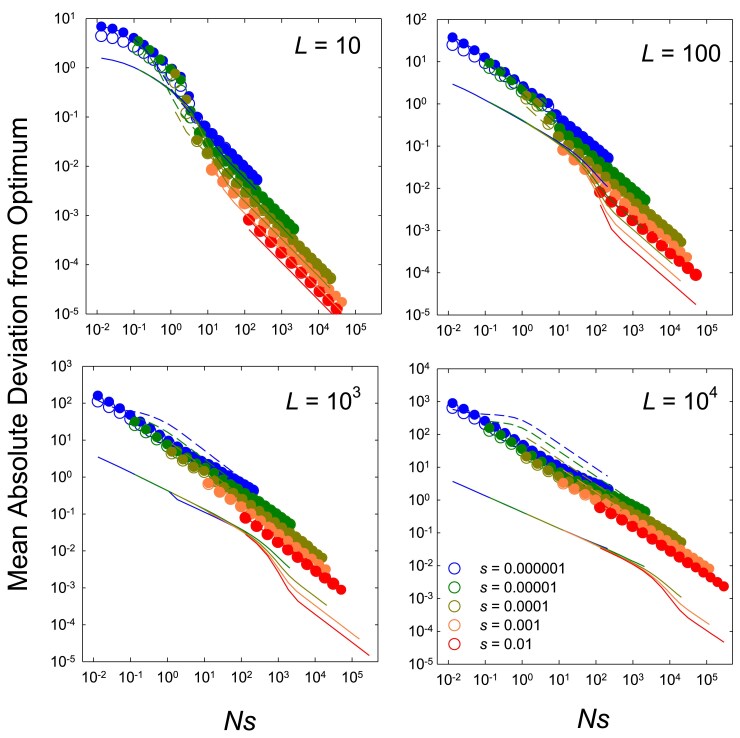
Average absolute deviation of phenotypic means from the optimum, $\bar{\left|\delta \right|}$, for the case in which the optimum is located at the extreme value of the genotype distribution (the half-Gaussian fitness function), $\theta_{S}=L$. Results from computer simulations are given for the case of no mutational bias (β=1, open points) and β=0.33 (closed points); except for Ns<1, these two sets of results are nearly indistinguishable. Solid lower lines, given for reference, are the theoretical results for an intermediate optimum with no mutational bias (from Fig. 1). Dashed lines are the analytical approximations given by Equation (13) shown for β=0.33 (and in most cases obscured by the closely fit data points).

Three striking patterns are revealed in Fig. 4 for the case of the half-Gaussian fitness function. First, relative to the situation in which $\theta_{S}=L/2$, the deviation of the mean from the optimum can be increased by one to two orders of magnitude. The effect is most pronounced when selection is not overwhelmingly strong and the number of sites is large (Ns<L/10). In effect, the hard reflecting boundary of the half-Gaussian reduces the width of phenotypic space within which a particular deviant can wander and remain in an elevated fitness state. Second, the effect of mutation bias on the mean deviation is very small unless Ns≪1, in which case the population means start to evolve in a nearly neutral fashion and hence are largely dictated by *β*. Third, the main determinants of the mean deviation are the selection-drift ratio *Ns* and the number of sites *L*, with an additional effect of *s* alone restricted to the deterministic domain, similar to what was found for the case of $\theta_{S}=L/2$ and β=1.

A rough analytical approximation to the mean deviation can be obtained by using the same approach that led to Equation (3), i.e. by weighting the distribution expected under neutrality, $\Phi_{n}(z)$, by $W{\left(z\right)}^{2N}$, and then normalizing to obtain the distribution expected under the joint influence of drift, mutation, and selection,


(10a) $$\Phi_{s}(z)=C\cdot \Phi_{n}(z)\cdot W{\left(z\right)}^{2N},$$

where *C* is a constant equal to the reciprocal of the sum of $\Phi_{n}(z)\cdot W{\left(z\right)}^{2N}$ over z=0 to *L*, which ensures that the probability density sums to 1.0, and


(10b) $$\Phi_{n}(z)=\frac{L!}{(L-z)!z!}{\left(\frac{\beta }{1+\beta }\right)}^{z}{\left(\frac{1}{1+\beta }\right)}^{L-z}$$

is the distribution of means under neutrality. Equation (10a) can be viewed as a multistate generalization of the Li–Bulmer model for the steady-state distribution of two-allele systems (Li 1987; Bulmer 1991), and similar expressions have been previously introduced in the context of quantitative genetics (Lande 1976; Barton 1989, 2017; Sella and Hirsh 2005; Barton and Coe 2009; and references therein). The expected mean phenotype resulting from fixations is then


(11) $$\mu (\bar{z})=\sum_{z=0}^{L}\Phi_{s}(z)\cdot z.$$

A more mechanistic explanation for the mean deviation in this case can be achieved by setting $\theta_{S}=L$ in Equation (3) and applying the expressions in Appendix B,


(12) $$[\theta_{S}-\mu (\bar{z})]≃\frac{L(1+\beta )}{4N_{e}sL\beta +(1+\beta {)}^{2}}≃\frac{1+\beta }{4N_{e}s\beta },$$

which unlike Equations (10)–(11), assumes a normal distribution of mean phenotypes. The approximation applies when $N_{e}sL\beta \gg 1$, and reduces further to $1/(2N_{e}s)$ in the absence of mutation bias, and to $1/(4N_{e}s\beta )$ when β≪1. Equation (12) generally yields results that are similar to those obtained with Equation (11), although the formulaic predictions of ${\bar{\left|\delta \right|}}_{1}$ using $N_{e}=N$ can often be substantially below the mean deviations observed in computer simulations.

To gain some insight into the degree to which $N_{e}$ is suppressed relative to *N*, one can equate Equation (12) to the observed mean deviation and solve for *ϕ*. Such analyses show that for the half-Gaussian fitness function, *ϕ* decreases monotonically with increasing *Ns*, increasing *L*, and decreasing *β*, diminishing to $10^{-3}$ in extreme cases (Supplemental Fig. 2). The estimates of *ϕ* can be predicted to a good degree of accuracy using the same formulae in Appendix E used for the case of an intermediate optimum, with one modification—letting the fraction of newly arising mutations having interference effects equal to $(1/2{)}^{1/\beta }$. This then yields estimates of *ϕ* in terms of the underlying model parameters ($u_{10}$, *β*, *L*, and *Ns*) that are generally within a factor of three relative to the observations and often only differing by a few percent.

There will again be an additional slight downward bias in the mean associated with segregating deleterious variants maintained by selection–mutation balance, noticeable only at very high *Ns*, and we find that this can be closely approximated by the use of a transitional expression as in Equations (7) and (8), in this case leading to an overall mean deviation from the optimum of


(13) $$\bar{\left|\delta \right|}≃(Lu_{10}/s)+[L-\mu (\bar{z})-(Lu_{10}/s)]e^{-2Ns},$$

where $Lu_{10}/s$ is the approximate number segregating deleterious mutations per genome in large populations, and $\mu (\bar{z})$ is estimated using Equation (12) with $N_{e}=\phi N$. The predicted results from Equation (13) are for the most part extremely close to the data obtained by computer simulations (dashed lines in Fig. 4), the only moderate exceptions involving large linkage blocks ($L>10^{3}$) and intermediate strengths of selection (0.1<Ns<100), where the mean deviation is overestimated.

Complications arise when the optimum is not as extreme as $\theta_{S}=L$, as the possibility then exists that the mean phenotype can wander above and below the optimum. If there is significant overlap between the distribution of means and $\theta_{S}$, it becomes essential to separately evaluate the mean deviations conditional on residing above and below $\theta_{S}$, as the absolute deviation is no longer equal to the mean deviation. An understanding of when this partitioning needs to be implemented can be achieved by considering the overall mean and standard deviation of the mean deviations from the optimum using Equations (3) and (4). Letting $\theta_{S}=xL$, Equation (12) generalizes to


(14a) $$[\theta_{S}-\mu (\bar{z})]=\frac{L(1+\beta )[x(1+\beta )-\beta ]}{4N_{e}sL\beta +(1+\beta {)}^{2}}≃\frac{\left(1+\beta )[x(1+\beta )-\beta \right]}{4N_{e}s\beta },$$

where the second approximation applies when $N_{e}sL\beta \gg 1$. This can provide an adequate entry for the first term of Equation (13) provided the distribution of mean phenotypes resides sufficiently far from $\theta_{S}$, say >2 standard deviations away. The standard deviation of the distribution of means is


(14b) $$\text{SD}(\bar{z})={\left(\frac{L\beta }{4N_{e}sL\beta +(1+\beta {)}^{2}}\right)}^{1/2}≃{\left(\frac{1}{4N_{e}s}\right)}^{1/2}.$$

Under a wide range of conditions, $\left[\theta_{S}-\mu (\bar{z})]/\text{SD}(\bar{z}\right)$ will be substantially smaller than two, in which case these expressions should be applied to Equation (D2) in the Appendix to obtain estimates of $\bar{\left|\delta \right|}$. As a first-order approximation, provided β<1, the additional load associated with segregating polymorphisms maintained by selection–mutation balance in large populations (Ns≫1), is


(15) $${\bar{\left|\delta \right|}}_{2}=\frac{Lu_{10}}{(1+\beta )s}.$$

As an example of the effects of less extreme $\theta_{S}$ than under the half-Gaussian, results are provided in Supplemental Fig. 3 for the case in which $\theta_{S}=3L/4$. These again show that the mean deviation from the optimum can be one to two orders of magnitude greater than that for the case of $\theta_{S}=L/2$ and β=1, that the effect of mutation bias is of second order relative to that of *Ns*, and that the theoretical predictions obtained with appropriate modifications of the methods noted above yield results that are satisfyingly close to those obtained by computer simulations.

### Distribution of site types

Finally, we consider the complications that arise when genomic sites differ in the magnitude of the selective effects associated with mutations. Such conditions are virtually certain to occur for most complex traits, as for example: (1) underlying amino-acid replacement sites will differ with respect to their contributions to core functions, protein folding, etc.; (2) silent sites will have varying influences on functions such as splicing of precursor mRNAs, folding of mature transcripts, and attractiveness to tRNAs; and (3) sites in noncoding regions will have variable effects on gene expression.

Multiple site types are expected to influence the evolutionary divergence patterns outlined above in three ways (Devi *et al*. 2023). First, a distribution of site types will extend the response of mean phenotypes to a wider range of population sizes, as sites with large effects will become fixed for favorable alleles at small *N*, whereas those with smaller effects will be rendered effectively neutral until population sizes have increased to the point where the strength of selection exceeds that of drift. Second, because selective interference is maximized among sites with mutations with identical fitness effects, distributing such effects across sites will alter the degree to which *ϕ* is reduced below 1.0, although each site type will have its own unique value of *ϕ* depending on its own abundance and that of adjacent types. An approximate rule of thumb is that if the ratio of selection coefficients between two site types (large and small) is $s_{L}/s_{S}$, from the perspective of large-effect sites, $(s_{L}/s_{S}{)}^{2}$ small-effect sites impose approximately the same amount of selective interference as one additional large-effect site (Devi *et al*. 2023). Finally, at any particular *N*, the overall level of selective interference will largely depend on the pool of sites with *s* in the vicinity of 1/N as sites with much larger effects will be essentially fixed for favorable alleles, whereas those with much smaller effects will be uninfluenced by selection.

**Fig. 5.**
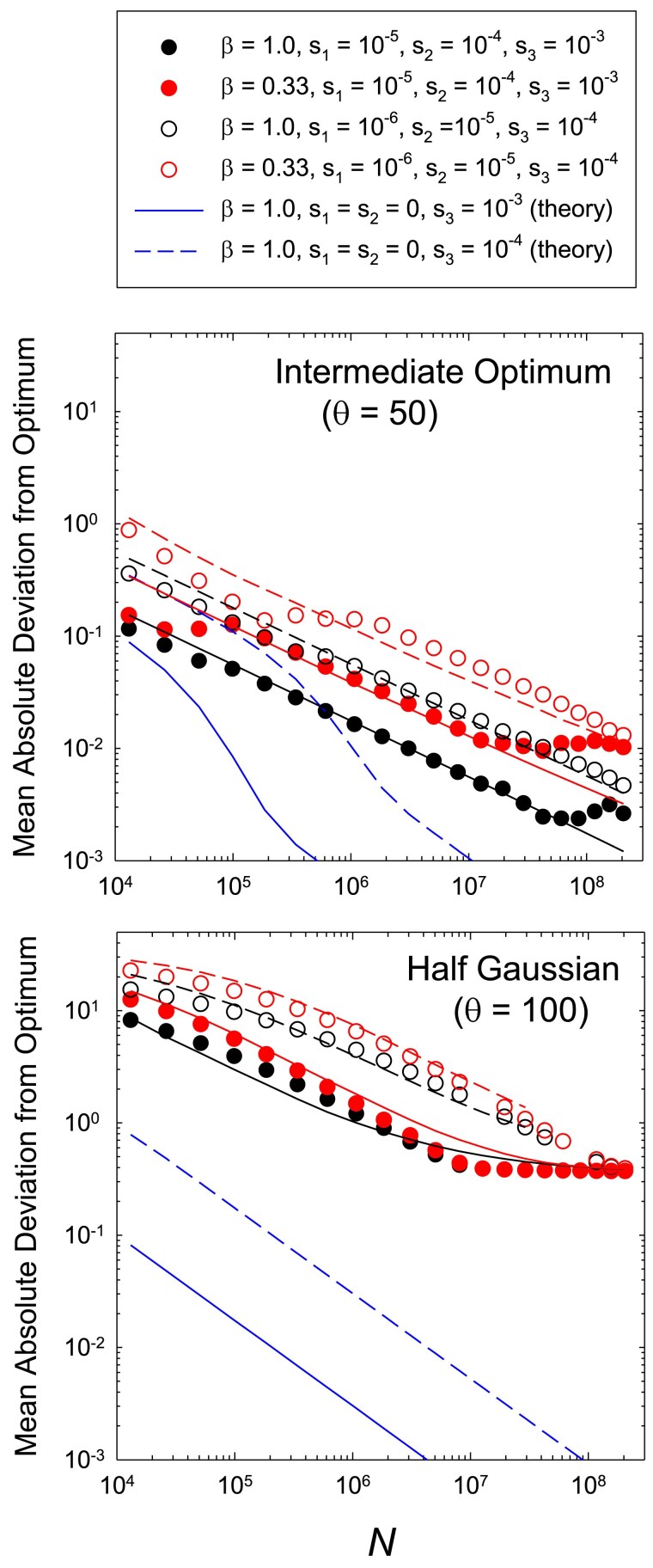
Average deviation of phenotypic means from the optimum for the case in which there are three types of sites, numbering $L_{3}=3333$, $L_{2}=333$, and $L_{1}=33$, with selection coefficients denoted in the legend. Results obtained by computer simulations are given for two levels of mutation bias for cases in which the optimum is intermediate ($\theta_{S}=L/2$) or at the extreme of the range of phenotypic variation ($\theta_{S}=L$). Performance is defined as $n_{1}+n_{2}(s_{2}/s_{1})+n_{3}(s_{3}/s_{1})$, where $n_{x}$ denotes the number of + alleles at the designated site type, which yields a maximum value of 100 when all sites are occupied by + alleles. The upper solid and dashed lines denote the expectations based on the theory outlined in the text. The two lower lines denote the theoretical expectations for the case of a single site type with no mutation bias.

Here, for illustrative purposes, we simply examine the case of an approximately negative exponential distribution of sites with three effects, arranged such that the pool of each of three effects contributes equally to overall performance, e.g. $L_{1}=33$ sites with s=0.001, $L_{2}=333$ sites with s=0.0001, and $L_{1}=3333$ sites with s=0.00001. Not surprisingly, the response of the mean deviation to the population size *N* is greatly diminished but also extended, relative to the case of single site types, owing to the progressive exit of site types from the realm of effective neutrality as *N* increases (Fig. 5). Roughly speaking, with the distribution of site types employed, each order of magnitude of increase in *N* opens up to selection a window of sites with an order of magnitude reduction in *s*. With the choice of site types used here, involving order-of-magnitude differences in *s*, there is little interference among sites, and some progress can be made in demonstrating how these more complicated scenarios might be dealt with.

For the case of a Gaussian fitness function with an intermediate optimum, the general approaches used above for single site types can be readily extended to obtain the mutual effects of all site types in an additive fashion. For example, for the situation in which there is no mutation bias (β=1) and the optimum for each site type *x* is $L_{x}/2$, Equations (6) to (8) can be used to estimate the total deviation associated with each site type, and the total mean absolute deviation from the optimum on the phenotypic scale is obtained as ${\bar{\left|\delta \right|}}_{\text{ major}}+({\bar{\left|\delta \right|}}_{\text{medium}}/10)+({\bar{\left|\delta \right|}}_{\text{minor}}/100)$. Here, the weightings are based on the 10-fold differences in the contributions of the different site types to the final phenotype, with the scale being set by the selection coefficient of the major site type (with the largest selection coefficient). When there is significant mutation bias, it again becomes necessary to calculate *ϕ*, the measure of the reduction in $N_{e}$ relative to *N*, and this needs to be done separately for each site type using Equations (E6a,b). Equations (3), (4), and (D2) are then used to obtain the mean deviation from the optimum for each site type associated with fixations, setting the additional small deviation associated with standing variation to $(1+\beta )u_{10}\sqrt{L_{x}}/s_{x}$ as in the single site-type case, and blending the two together using $e^{-2Ns_{x}/L_{x}}$. The total influence of all three site types is then obtained using the weighted sum noted above.

As can be seen in the upper panel of Fig. 5, these approaches yield good first-order approximations to the results obtained by simulation. The same approach should work for a distribution of effects with more finely spaced categories, but if adjacent categories have similar enough selection coefficients so as to cause mutual interference, these additional effects would need to be incorporated by increasing the counts of effectively interfering sites using the squared weighting scheme noted above. Supposing there are $n_{x}$ sites with selection coefficient $s_{x}$, if for the next closest site type (*y*), $n_{y}(s_{x}/s_{y}{)}^{2}\ll n_{x}$, then mutual interference can safely be ignored.

Things are less straightforward when the optimum is located at the end of the range of possible phenotypic variation. As anticipated in the prior section, the magnitude of deviation from the optimum is inflated in this case, as the scaling of phenotypic changes with biologically discernible differences (greater than a few percent of the phenotypic range) extends over several orders of magnitude of *N*. Again in this particular example, as the three effects employed are far enough apart that they cause minimal interference with each other, it is possible to sum the results noted above for the half-Gaussian fitness function. This first requires estimates of *ϕ* for each effect (each with its values of *L* and *s*), obtained by the approaches outlined in the previous section, and then the category-specific deviations are obtained with Equation (12). Using Equation (13), the deviation associated with standing variation is further incorporated, and then


(16) $$\bar{\left|\delta \right|}≃\theta_{S}-\frac{s_{1}(L_{1}-{\bar{\left|\delta \right|}}_{1})-s_{2}(L_{2}-{\bar{\left|\delta \right|}}_{2})-s_{3}(L_{3}-{\bar{\left|\delta \right|}}_{3})}{s_{1}},$$

where ${\bar{\left|\delta \right|}}_{x}$ now refers to the total deviation associated with site-type *x*. There is one final nuance for this analysis—as each category of site contributes only partially to total trait performance, the upper limit to performance shifts as the population size increases and opportunities for selective advance of mutations with smaller effects (which were previously kept at mutation balance) are opened up. This causes a reduction in the effective strength of selection typically operating on sites, and we have found that using s/10, rather than *s*, as a measure of selection intensity yields respectable fits to the data (again for the particular cases outlined in Fig. 5), whereas the use of *s* does not.

Again, although some aspects of these analyses are not formally rigorous, the fits to the simulated data are seen to be reasonably good in the lower panel of Fig. 5. This suggests that the general approaches being used capture the essence of the overall process, and may be useful starting points for future attempts to explore the influences of alternative distributions of site types. The more significant issue is the difficulty of achieving empirical insight into the latter. For complex traits, there are reasons to expect such distributions to be strongly skewed to sites with smaller effects, as employed above (Walsh and Lynch 2018; Lynch 2024), although this is ultimately an empirical issue. (In the illustrated examples in Fig. 5, $L_{1}=33$, $L_{2}=333$, and $L_{3}=3333$, with two sets of $s_{x}$ used such that $L_{x}s_{x}$ remains constant across categories, i.e. each category of sites contributes equally to a trait with maximum value 100).

## Discussion

Owing to random genetic drift and recurrent mutation, no character can evolve to an absolute state of perfection, although very large populations may come close barring the additional matters of biophysical constraints and the baseline load of recurrent deleterious mutations. Drift barriers, which are universal properties of all finite populations (i.e. all of biology), denote the limits to which mean phenotypes are expected to wander over evolutionary time, with their exact positions depending on the strength of selection relative to drift and on the degree of mutation bias. However, population mean phenotypes do not simply evolve to drift barriers. Rather, they wander within a particular range set by the latter. For example, with purely directional selection for larger phenotypes, the upper bound to which the mean phenotype evolves represents the position above which further refinements cannot be advanced by selection, whereas the lower bound represents the position below which further descent is readily opposed by selection. In the case of a fitness function with an optimum intermediate to the range of attainable genotypic values, two drift barriers straddle the optimum, but in this case the optimum is at least transiently accessible.

An understanding of the limited reach of selection can aid in the interpretation of comparative phenotypic data among species, in particular the degree to which these may be compatible with alterations associated with shifts in baseline population-genetic parameters (e.g. power of drift, recombination, and mutation bias) rather than consequences of adaptive divergence. As population mean phenotypes are free to wander within the confining drift-barrier limits, over time a steady-state distribution of mean phenotypes can be expected to arise, provided the population-genetic parameters and underlying genetic architecture of the trait remain unchanged. Under this view, with all features remaining constant except the effective population size, gradients of mean phenotypes with respect to changes in $N_{e}$ can be expected under a regime of persistent directional selection (Lynch 2018, 2020; Devi *et al*. 2023). However, as shown here, this is also true in the case of stabilizing selection, provided the equilibrium mean phenotype under mutation alone differs from the optimum, which is likely nearly always the case. That is, mutation bias at the molecular level is not essential to the generation of gradients of mean phenotypes, although such bias can sometimes magnify the degree of such variation.

Here, we have examined some of the nuances that arise with a Gaussian (bell-shaped) fitness function, which naturally stabilizes phenotype distributions as the strength of selection progressively increases with greater deviations from the optimum. This work complements prior work on a form of pure directional selection, the exponential fitness function (Devi *et al*. 2023). Through the combined use of computer simulations and development of analytical approximations for the population-genetic outcomes, several significant features have been revealed with respect to the deviation of mean phenotypes from their optimum values.

First, for the special case of an optimum exactly intermediate in the range of possible genotypic values (L/2) and no mutation bias (β=1), the steady-state distribution of mean phenotypes is symmetrical about the optimum, which also coincides with the neutral expectation. In this case, although the grand mean phenotype coincides with the optimum regardless of the population size (*N*), the width of the distribution increases with decreasing *N*, reducing the average performance of the trait. Under this ideal setting, commonly employed in quantitative-genetic theory, the absolute deviation of the mean phenotype from the optimum decreases with $N_{e}s$, a measure of the strength of selection relative to that of drift, and increases with the number of genomic sites within linkage blocks (*L*) (Fig. 1), which defines the level of selective interference among simultaneously segregating mutations, as shown in Equation (6).

Second, when the optimum deviates from L/2, even with no mutation bias, there is a directional pull on the mean phenotype because the optimum does not coincide with the expectation resulting from mutation alone. The distribution of mean phenotypes is then no longer symmetrical about the optimum, and the mean deviation is increased relative to the expectation under the ideal symmetrical model. For the case in which the optimum is located at one end of the realizable range, so that selection is purely directional, the mean deviation from the optimum can be up to two orders of magnitude greater than when the optimum is intermediate. For even more extreme situations in which the optimum resides well outside of the range (0,L), we expect the results to converge on those already found for an exponential fitness function (Devi *et al*. 2023), as the tail of the Gaussian function is approximately exponential in form.

As there is no reason to think that the distribution of genotypes governed by mutation alone will have a mean that exactly coincides with the optimum phenotype, these kinds of effects are likely to be biologically general. Similar points have been made before with different models for quantitative traits under Gaussian selection (Waxman and Peck 2003; Zhang and Hill 2008; Charlesworth 2013), where it has been noted that the incorporation of mutation bias can lead to quite different predictions than in prior models that assume mutational effects to be unconditionally and symmetrically distributed around the current mean phenotype (e.g. Bürger and Lande 1994). Unlike the current study, all of these models assume free recombination, no linkage disequilibrium, and an optimum well-embedded in the range of phenotypic variation. The model of Charlesworth (2013) is closest to ours, as it employs biallelic loci and finite population sizes, whereas the others utilize a continuum-of-allele framework, which can yield some unusual results. For example, Waxman and Peck (2003) assume an infinite population size, which leads to a peculiar situation in which high mutation bias brings the mean phenotype closer to the optimum because most mutations are so extreme as to be effectively lethal.

Third, there are three general domains in the response of the deviation of mean phenotypes from the optimum, $\bar{\left|\delta \right|}$, to shifts in population size. For *Ns* progressively declining below 1.0, the mean phenotype converges on the expectation under effective neutrality, and $\bar{\left|\delta \right|}$ approaches a constant maximum value. For 0.1<Ns<L/20, the absolute mean deviation typically declines as an approximate power-law relationship with *Ns*, as the mean phenotype asymptotically approaches the optimum. Finally, as *Ns* exceeds L/20, the system behaves in an essentially deterministic fashion, with drift no longer playing a role, and the small deviation from the optimum being solely dictated by the recurrent introduction of deleterious mutations.

Fourth, although analytical expectations can be developed for the behavior of the mean phenotype in terms of the absolute population size *N*, for most situations these expressions have to be modified to incorporate an effective population size $N_{e}$, which can be well below N/100 depending on the size of the linkage block, magnitude of mutation bias, and strength of selection. Unfortunately, the $N_{e}$ associated with the drift of mean phenotypes need not be closely related to the $N_{e}$ dictating the maintenance of variation within populations, which is generally fairly close to the *N* used in the preceding formulations. This raises challenges in the application of theory to data, as extrapolation from silent-site variation is the primary method for obtaining empirical estimates of effective sizes of natural populations (Walsh and Lynch 2018). Heuristic first-order approximations have been obtained for the $N_{e}$ associated with the drift of mean phenotypes (Appendix E), although more rigorous derivations are needed.

Moreover, as illustrated in Fig. 3, the same reduction in $N_{e}$ relative to *N* can be obtained with many different combinations of *s*, *Ns*, *β*, and *L*. Notably, the influence of mutation bias on the drift $N_{e}$ is nonmonotonic, with the maximum reduction occurring when β=0.33. The key point here is that although Ns<1 is often taken to be the approximate benchmark below which drift starts to have a substantial impact on evolution and standing variation, this approximation becomes increasingly unreliable when sites are linked, mutation is directionally biased, and multiple site types are simultaneously segregating. As similar points have been made previously by Good *et al*. (2014) using a very different model involving unconditionally deleterious and unbiased mutation, these concerns seem to be quite general. This raises significant concerns for studies that attempt to infer historical patterns of selection and drift from measures of standing variation.

Finally, we note that aside from allowing the fitness function to be truncated on one side, we have only considered the situation in which the decline in fitness around the optimum is symmetrical. Some attempts have been made to evaluate how asymmetrical (skewed) fitness functions can lead to the evolution of mean phenotypes deviating from the optimum and more towards the shoulder of the fitness function (Vercken *et al*. 2012; Urban *et al*. 2013). Although an absence of mutation bias was apparently assumed in both studies, as noted above, there will be some effect owing to the displacement of the mean from the optimum. Thus, an extension of this work to include varying levels of mutation bias could provide interesting insight into how this might interact with asymmetrical selection to produce more- vs. less-pronounced deviations of mean phenotypes from their optima, and help identify ways to determine the extent to which apparently maladapted phenotypes are a consequence drift, mutation bias, and/or asymmetrical fitness functions.

The essential concluding point from the preceding results and from prior analyses (e.g. Lynch 2018, 2020; Devi *et al*. 2023) is that differences in $N_{e}$ alone can cause observable gradients in the mean phenotypes of populations experiencing identical selection and mutation pressures. The degree to which such scaling relationships can be detected and interpreted in broad phylogenetic comparisons depends on numerous factors, including the form of the fitness function, the distribution of genomic site types, the degree of mutational bias, and the strength of recombination. Nonetheless, the fact that power-law relationships relating mean phenotypes to *N* are predicted under plausible distributions of mutational effects and constant forms of selection raises issues with respect to the interpretations of studies in evolutionary allometry commonly focused on such bivariate regressions. This is a concern because the explanatory variable (on the *x* axis) in such studies is almost always a measure of body size, which in a broad phylogenetic context scales negatively with *N*. Yet, almost all studies in evolutionary allometry focus entirely on explanations based on adaptive tradeoffs or physical constraints, and leave no room for involvement of population-genetic processes.

Of course, the strength and pattern of selection operating on many traits, particularly those relating to external ecological factors, can vary widely among species for multiple reasons, thereby obscuring any anticipated effects of drift, which may be secondary. However, this may be less of a problem for intracellular features with conserved functions across the Tree of Life, and the drift-barrier hypothesis has been invoked to explain a diversity of evolutionary patterns involving such traits, including mutation rates, ages at senescence, strengths of transcription-factor binding sites and interfaces within multimeric proteins, and distributions of phosphorylation sites (reviewed in Lynch 2024).

One of the most compelling examples of the limitations of trait evolution by a drift barrier is revealed by ∼100 genome-wide estimates of mutation rates, which scale strongly negatively with measures of the coalescent $N_{e}$, the interpretation being that persistent downward directional selection on error rates is progressively thwarted by drift in populations with diminished $N_{e}$ (Lynch *et al*. 2023). However, drawing from observations on laboratory constructs of yeast, Liu and Zhang (2021) argued for a rejection of this hypothesis in favor of one invoking stabilizing selection alone. Here, we take the opportunity to outline some of the practical difficulties in using comparative data to infer the operation of specific forms of selection, e.g. directional vs. stabilizing, and the interactive role played by random genetic drift.

First, as demonstrated above, the operation of stabilizing selection is not incompatible with the functional role of a drift barrier, as such constraints exist under virtually any fitness function. Moreover, when the expectations of mean phenotypes under mutation alone are not aligned with the optimum under stabilizing selection, populations may effectively behave as though they are evolving directionally even though the fitness function is stabilizing. Second, as noted above, the mean phenotypes of populations are not expected to reside specifically at a drift barrier, even under purely directional selection. Rather, the latter is best viewed as a reflecting boundary, above which there is a range within which the mean phenotype is able to wander in an effectively neutral manner, which in some cases can be considerable (Lynch 2011, 2018). For this reason, although theory may allow qualitative statements on the scaling of mean phenotypes with respect to $N_{e}$, quantitative statements about the precise locations of drift barriers require information on the distribution of fitness effects of new mutations, degree of linkage, and mutation bias, none of which is easily acquired. Third, an ability to produce favorable mutant phenotypes beyond a supposed drift barrier does not negate the latter’s existence, as the key issue is whether natural selection’s ability to promote such mutations is compromised. Finally, one might think that a comparison of standing levels of variation of a trait with the neutral expectation would be revealing as to the form of selection and the degree to which a trait mean is close to a drift barrier. However, the level of genetic variance maintained in a population under the joint influence of drift and selection is not equivalent to that expected under a neutral model (Walsh and Lynch 2018; Devi *et al*. 2023), and depression of the genetic variance relative to neutrality is incapable of distinguishing between models of directional vs. stabilizing selection (Barton 1989).

These caveats raise significant challenges in connecting observational data with drift-barrier theory. Drift barriers must be universal to the evolution of all traits across the Tree of Life, just as gravity is a universal physical force. However, the degree to which differences in the magnitude of random genetic drift translate into substantial lineage-specific phenotypic differences, including the scaling relationships of phenotypes with $N_{e}$ (should the appropriate measure of the latter even be obtainable), is a matter of the genetic architecture of the trait under consideration as well as the ability of the investigator to make precise measures in reasonably controlled environmental settings. There remains a need for further theoretical work in this area, but this should ultimately be guided by the acquisition of key information on the links between genotypes, phenotypes, and fitness of diverse organisms experiencing substantially different population-genetic environments.

## Supplementary Material

iyaf031_Supplementary_Data

## Data Availability

The authors affirm that all data necessary for confirming the conclusions presented in the article are represented fully within the article and figures. The C++ code for the simulation data can be found at https://github.com/LynchLab/Asexual-Gaussian-Selection). Supplemental material available at GENETICS online.

## Appendix A: Local strength of selection with the Gaussian fitness function

With a fitness function defined by Equation (2), although there is a single parameter describing the overall strength of selection (*s*), only when the deviation from the optimum $\delta =\bar{z}-\theta_{S}=1$ is the strength of selection on a haplotype essentially equal to *s*, as this leads to a reduction in fitness from the optimum of $1-e^{-s}≃s$, assuming s≪1. However, as the curvature of the Gaussian selection function is a function of |δ|, to understand the strength of selection separating adjacent haplotypes, we require their relative fitness difference,

(A1 )
$$s^{*}=\frac{W(\delta )-W(\delta +1)}{W(\delta )}=1-e^{-s(2\delta +1)}.$$

This shows that as a parent allele deviates further and further from the optimum, the relative reduction in fitness of the next worst allele increases. This measure is of significance, as we expect the power of drift to compete with the strength of selection only up to the point at which $1/N>s^{*}$. For a population stalled at mean phenotype $\bar{z}$, for sδ≪1, the effective strength of selection for a beneficial mutation (moving the mean closer to $\theta_{s}$) is $s^{*}≃2s(\bar{z}-\theta_{s})$.

## Appendix B: Diffusion approximation with an intermediate optimum

Using a quantitative genetics approach for the case of Gaussian selection combined with reversible mutation of *L* equivalent biallelic sites and with random genetic drift, Lynch (2018) found that the steady-state distribution of mean phenotypes under drift-mutation-selection balance, $\left(\bar{z}\right)$, is Gaussian with mean and variance, respectively,

(B1 )
$$\mu (\bar{z})=\frac{\kappa \theta_{S}+\theta_{N}}{\kappa +1},$$

(B2 )
$$\sigma^{2}(\bar{z})=\frac{\sigma_{N}^{2}}{\kappa +1},$$

where $\theta_{S}$ and $\theta_{N}$ are the expected means of the distributions in the presence of strong selection and complete neutrality, the first being the optimum phenotype (on the scale of *L*, under the assumption of additive genetic effects), and

(B3 )
$$\theta_{N}=\frac{Lu_{01}}{u_{01}+u_{10}},$$

where *L* is the number of sites, and $u_{01}$ and $u_{10}$ are the rates of forward (− to +) and reverse (+ to −) mutation. In addition,

(B4a)
$$\sigma_{N}^{2}=\frac{\sigma_{M}^{2}}{2N(u_{01}+u_{10})}$$

is the variance among means under neutrality, with *N* being the population size, and $\sigma_{M}^{2}$ being the within-population genetic variance (sum of expected heterozygosities over all sites) under the assumption of mutation-drift equilibrium,

(B4b)
$$\sigma_{M}^{2}=\frac{4NL\beta u_{10}}{1+\beta +Nu_{10}(1.0+6\beta +\beta^{2})}≃\frac{4NL\beta u_{10}}{1+\beta },$$

with mutational bias $\beta =u_{01}/u_{10}$, and the approximation applying for $Nu_{10}\ll 1$, which is the case for all conditions in evaluated in this paper. Finally, letting

(B5 )
$$\sigma_{S}^{2}=\frac{(1/2s)+\sigma_{G}^{2}}{2N_{e}},$$

where $N_{e}$ is the effective population size, be the variance of means when selection is the prevailing force, with $\sigma_{G}^{2}$ being the equilibrium within-population genetic variance,

(B6 )
$$\kappa =\frac{\sigma_{N}^{2}}{\sigma_{S}^{2}}.$$

Lande (1976) obtained a less general result than that above; by assuming an absence of mutational bias (with mean mutational effect equal to zero) and negligible mutational variance relative to standing variation, he derived an asymptotic Gaussian distribution of mean phenotypes with an overall mean of $\theta_{S}$ and variance given by Equation (B5).

Two key issues in the above derivations are the definitions of the genetic variance and the effective population size used in Equation (B5). For situations in which the mean is close to the optimum, an approximation for $\sigma_{G}^{2}$ can be obtained by assuming that most of the sites are fixed for the appropriate numbers of + and − alleles, and that genetic variation is maintained by the balance between recurrent mutation and selection. For example, if the optimum is in the center of the range of possible haplotypes, $\theta_{S}=L/2$, then the numbers of the two types of alleles will be in equal abundances within most haplotypes, with half mutating at rate $u_{10}$ and the other half mutating at rate $\beta u_{10}$, which with a fractional removal equal to *s* in both cases per generation, yields

(B7 )
$$\sigma_{G}^{2}≃\frac{(L/2)(1+\beta )u_{10}}{s+(1+\beta )u_{10}}≃\frac{L(1+\beta )u_{10}}{2s},$$

where the second approximation applies when mutation is weak relative to selection. Both approximations work well when the mean deviates less than 1% from the optimum, but otherwise can overestimate the observed genetic variance by several fold. In any event, in comparing Equation (B7) with the first term in Equation (B5), it is clear that the total haplotype mutation rate must be >0.1 or so for the genetic variance term to make a meaningful contribution to the numerator, suggesting that as a first-order approximation, $\sigma_{S}^{2}≃1/(4N_{e}s)$.

## Appendix C: Analytical approximation of the absolute mean deviation from an intermediate optimum, assuming unbiased mutation

The preceding results can be distilled down to a relatively simple expression for the behavior of $\bar{\left|\delta \right|}$ when the mutation rates between the two alleles are directionally unbiased, i.e. $u_{10}=u_{01}=u$, β=1.0, and $\theta_{N}=L/2$. From Equation (B4b), the equilibrium genetic variance under neutrality reduces to

(C1a)
$$\sigma_{M}^{2}=\frac{2NLu}{1+4Nu}≃2NLu,$$

the approximation applying when Nu≪1, and from Equation (B4a),

(C1b)
$$\sigma_{N}^{2}=\frac{\sigma_{M}^{2}}{4Nu}=\frac{L}{2(1+4Nu)}≃\frac{L}{2}.$$

Further using the approximation, $\sigma_{S}^{2}=1/(4N_{e}s)$, from Equation (B6),

(C2 )
$$\kappa =\frac{\sigma_{N}^{2}}{\sigma_{S}^{2}}≃2N_{e}Ls.$$

Because it is assumed here that there is no mutational bias, the grand mean phenotype coincides with the optimum, $\theta_{S}$, and from Equation (B2), the variance of means is

(C3 )
$$\sigma^{2}(\bar{z})≃\frac{L}{2(2N_{e}Ls+1)},$$

the approximation again assuming weak mutation. The mean absolute deviation from the optimum is then simply the scaled standard deviation,

(C4 )
$$\bar{\left|\delta \right|}=\sqrt{2\sigma^{2}(\bar{z})/\pi }.$$

## Appendix D: Mean absolute deviation of means from the optimum

Given that our focus is on the efficiency of selection, when a fitness function has a stabilizing component, there is a need for a measure of the *absolute* deviation from the optimum, as the steady-state distribution of means may straddle the optimum. With a Gaussian fitness function, the solution is straightforward under two conditions. First, if the steady-state distribution is much more than two standard deviations from the optimum, the mean deviation is closely approximated by the difference between the overall mean and the optimum. Second, if the overall mean coincides with the optimum, and the distribution is Gaussian, the mean absolute deviation is $\sigma \sqrt{2/\pi }$. More generally, we need to determine the fractions of the distribution above and below the optimum, and the means of these two portions of the distribution relative to the truncation point.

Following the results from the preceding section, we assume that the distribution of means, $\varphi (\bar{z})$, is Gaussian with mean *μ* and variance $\sigma^{2}$, e.g. as given by Equations (B1) and (B2). Denoting the optimum phenotype as $\theta_{S}$, the desired measure is

(D1a)
$$\bar{\left|\delta \right|}=\int_{-\infty }^{\infty }|\bar{z}-\theta_{S}|\varphi (z)\;dz,$$

which expands to

(D1b)
$$\begin{array}{rl} \bar{\left|\delta \right|}\;= & \frac{1}{\sqrt{2\pi \sigma^{2}}}[\int_{\theta_{S}}^{\infty }(\bar{z}-\theta_{S})\exp\;\left(-\frac{(\bar{z}-\mu {)}^{2}}{2\sigma^{2}}\right)d\bar{z}\\ & \;-\int_{-\infty }^{\theta_{S}}(\bar{z}-\theta_{S})\exp\;\left(-\frac{(\bar{z}-\mu {)}^{2}}{2\sigma^{2}}\right)d\bar{z}]. \end{array}$$

This can be shown to be

(D2 )
$$\bar{\left|\delta \right|}=\sigma \sqrt{\frac{2}{\pi }}\exp\;\left(-\frac{(\theta_{S}-\mu {)}^{2}}{2\sigma^{2}}\right)+(\theta_{S}-\mu )\;\text{erf}\left(\frac{\theta_{S}-\mu }{\sigma \sqrt{2}}\right),$$

where erf denotes the error function. Consistent with the verbal argument given above, when $\theta_{S}=\mu$, Equation (D2) simplifies to

(D3a)
$$\bar{\left|\delta \right|}=\sigma \sqrt{\frac{2}{\pi }},$$

and when $\left|\theta_{S}-\mu \right|$ is large relative to *σ*, it converges to

(D3b)
$$\bar{\left|\delta \right|}≃|\theta_{S}-\mu |.$$

## Appendix E: Reduction in effective population size resulting from selective interference between competing favorable mutations

If a population finds itself slightly off a selective optimum, then mutations moving haplotypes back towards the optimum will be selectively favored. However, in sufficiently large populations, multiple secondary mutations will arise prior to the fixation of the first such mutation otherwise destined to fixation. This then diminishes the efficiency of selection, as only one mutation can fix at a time, thereby reducing the fixation effective population size.

There are two ways by which secondary mutations can influence the sojourns of beneficial mutations. Lineage interference occurs when another competing beneficial arises in a lineage outside of that containing the target mutation, inhibiting the latter from going to fixation, given that the competing lineages are equivalent in fitness. Lineage contamination occurs when secondary deleterious mutations arise in the target lineage, impeding progress towards fixation. In the following, we focus only on lineage interference, as secondary deleterious mutations will occur in both the target and off-target lineages.

To obtain an approximation for the interference effect, we follow the logic outlined in Devi *et al*. (2023), denoting the effective population size as $N_{e}=\phi N$, with

(E1a)
$$\phi =\frac{1}{1+I},$$

being the reduction in the effective population size resulting from *I* effectively competing parallel mutations arising while the target beneficial mutation is otherwise en route to fixation. Given the complexity of the issues, the approach we take is heuristic, not a derivation from first principles, But as will be shown, the resultant expressions provide good approximations to the behavior of *ϕ* in relation to the underlying genetic parameters, and therefore seem to capture the essence of interference processes.

First, we require information on the mean time to fixation of a beneficial mutation (conditional upon fixation), *τ*, as this determines the length of time during which the target mutation is vulnerable to interference. As pointed out by Charlesworth (2020), numerous expressions have been suggested for *τ* as a function of $N_{e}$, some of which give nonsensical values in certain domains of $N_{e}s$. An expression from Charlesworth (2022) is useful in the weak selection regime,

(E2a)
$$\tau =2\phi N\left(1-\frac{(\phi Ns{)}^{2}}{18}\right),$$

and we rely on this until it breaks down when ϕNs>1. A derivation of Gale (1990), modified for haploids,

(E2b)
$$\tau ≃\frac{3.927+2\ln\;(\phi Ns/2)}{s}-2\phi -\frac{2}{Ns},$$

is reasonably suitable for $N_{e}s\gg 1$. The transition between the two is relatively seamless from the standpoint of the current analyses.

Second, we must account for the number of potentially competing mutations, denoted here as $I_{p}$. One way to approach the problem is to assume that selection is strong enough that the population typically finds itself nearly fixed for a mean phenotype just one unit above or below the optimum. Supposing the population finds itself one position below the optimum, $\theta_{S}$ (which is on the scale of *L*), then $\beta u_{10}(\theta_{S}-1)≃\beta u_{10}\theta_{S}$ is the rate of origin of new competing (upward) mutations per individual in nontarget lineages, with $\beta u_{10}$ being the rate of origin of new competing (upward) mutations per individual site in the nontarget lineage, and the approximation assuming large *L* (justified by the fact that interference will be minimal with small *L*). Given that the target lineage has an average population size of ≃N/2 during its sojourn from a frequency of 1/N to 1.0 (assuming it is destined to fixation), this implies a total of $\beta u_{10}N\theta_{S}/2$ potentially competing beneficial mutations. Alternatively, should the population find itself one position above the optimum, there would be $u_{10}N(L-\theta_{S})/2$ potentially competing (downward) beneficial mutations. As these two starting points have approximate probabilities of 1/(1+β) and β/(1+β), respectively, the predicted total number of interfering mutations per target event is

(E3a)
$$I_{p}≃\frac{\beta u_{10}NL}{2(1+\beta )}.$$

An alternative approach to the problem is to assume that mutation bias is strong enough that the full distribution of genotypes is to one side of the optimum, so that all of the mutations in the appropriate direction (here assumed to upward, given the downward mutation pressure), regardless of genetic background, are beneficial. If it is further assumed that selection is strong enough to keep the population mean sufficiently close to the optimum that $(L-\theta_{S})/L$ is the average fraction of sites occupied by − alleles, then

(E3b)
$$I_{p}≃\frac{\beta u_{10}N(L-\theta_{S})}{2}.$$

For an intermediate optimum, $\theta_{S}=L/2$ and no mutation bias (β=1), the two approaches give identical results. If $\theta_{S}=L/2$ (intermediate optimum), the first approach predicts more interference if β<1. If β<1 and the optimum is intermediate or larger, the first approach always gives more interference.

Third, only a fraction of competing mutations is destined to fixation, which we define as the standard fixation probability of a new mutation with fitness benefit *s*,

(E4a)
$$p_{f}=\frac{1-e^{-2N_{e}s^{*}/N}}{1-e^{-2N_{e}s^{*}}}$$

(Kimura 1983). The relevant selection coefficient here depends on the distance of the genotypic mean from the optimum, with differentiation of the fitness function showing that

(E4b)
$$s^{*}≃2s(\theta_{S}-\bar{z}),$$

assuming $\theta_{S}>\bar{z}$. This expression also needs to be substituted for *s* in the expressions for *τ*. However, a circularity arises here in that $\bar{z}$ depends on *ϕ* and cannot be predicted in advance. To circumvent this issue, we assume that the mean phenotype is positioned at the neutral expectation, such that $\bar{z}=\theta_{N}=L\beta /(1+\beta )$, which necessarily means that we are overestimating the prevailing strength of selection. We will make further use of the approximation

(E4c)
$$p_{f}=\frac{2\phi s^{*}}{1-e^{-2\phi Ns^{*}}},$$

given that the numerator term is generally ≪1.

Fourth, we suggest that the strength of selection operating on a mutation must be on the order of the magnitude of genetic drift or greater if it is to compete for fixation, and to allow for this, we use the weighting term $N_{e}s^{*}/(1+N_{e}s^{*})$, which asymptotically approaches zero as $N_{e}s^{*}\to 0$ and 1.0 as $N_{e}s^{*}\to \infty$.

Fifth, owing to the presence of segregating mutations in the population, there is an additional factor *k* to account for the fact that not all mutations in the nontarget lineage are positioned in the full haplotype distribution such that they equal or surpass the target mutation in terms of fitness. The idea here is that secondary mutations arising on haplotypes with phenotypes further from the optimum than the mean phenotype will typically be destined to loss and hence irrelevant to the fixation process, whereas those arising on haplotypes closer to the optimum will compete with the target mutation for fixation. (When examining selection under an exponential fitness function, Devi *et al*. (2023) found that k≃1/4 serves as a decent first-order approximation. Here, we find by inspection that k≃1/32 provides an optimal fit of predicted to observed *ϕ* from computer simulations involving an intermediate optimum). This simple treatment assumes that *k* is independent of all other genetic parameters. In the future, it will be desirable to have theory that predicts *k* from first principles, and this presumably will be a function of the average form of the haplotype distribution.

Taking all of these terms together,

(E5 )
$$I≃\tau \cdot I_{p}\cdot p_{f}\cdot \frac{N_{e}s^{*}}{1+N_{e}s^{*}}\cdot k.$$

Substituting and rearranging from Equation (E1), and further expanding terms, leads to the transcendental equation,

(E6a)
$$1=\phi \left\{1+\left(\tau \cdot I_{p}\cdot \frac{2\phi s^{*}}{1-e^{-2\phi Ns^{*}}}\cdot \frac{\phi Ns^{*}}{1+\phi Ns^{*}}\cdot k\right)\right\}.$$

Equation (E6a) can be used to solve for *ϕ* by standard optimization procedures, noting that *τ*, as defined in Equations (E2a,b), is also a function of *ϕ*.

Owing to its multiple terms, Equation (E6a) is not very revealing with respect to the relative contributions and scaling relationships of the underlying factors influencing *ϕ*. However, if $2\phi Ns^{*}<1$, the first term in the denominator can be approximated as $2\phi Ns^{*}$, and Equation (E6a) can be written as a polynomial equation,

(E6b)
$$1≃\phi (1-Ns^{*})+\phi^{2}(Ns^{*})+\phi^{3}(2I_{p}kNs^{*})-\phi^{5}\left(\frac{I_{p}k(Ns^{*}{)}^{3}}{9}\right),$$

where Equation (E2a) has been used for *τ*.

This approximation can be further simplified in special cases. In particular, because ϕ<1, if $Ns^{*}<1$, the final term is small relative to the third term on the right (this is also equivalent to assuming that τ≃2ϕN). In addition, because $I_{p}$ is a linear function of *L*, in the limit of large *L* the first two terms on the right become of negligible importance. Recalling the definition of $s^{*}$ from Equation (E4b), then yields the approximation (for large *L* and weak selection),

(E7a)
$$\phi ≃{\left(\frac{1}{2I_{p}kNs(\theta_{S}-\bar{z})}\right)}^{1/3}.$$

Note that because $I_{p}$ scales linearly with *NL* and $\left(\theta_{S}-\bar{z}\right)$ is expected to scale with *L*, this expression suggests that *ϕ* should scale with $[(LN{)}^{2}s{]}^{-1/3}$. There remains the problem of the term $\left(\theta_{S}-\bar{z}\right)$ in $s^{*}$, as this is expected to be a function of other model parameters, including *N* and *ϕ*. If, however, we take $\left(\theta_{S}-\bar{z})≃(\theta_{S}-\theta_{N})=L(1-\beta )/[2(1+\beta )\right]$ as a rough approximation (but overestimate), some further progress can be made, especially keeping in mind that use of an appropriate value for the scaling parameter *k* might accommodate this overestimation. Further using Equation (E3a) to define $I_{p}$, Equation (E7a) becomes

(E7b)
$$\phi ≃{\left(\frac{(1+\beta {)}^{2}}{\beta (1-\beta )}\cdot \frac{1}{u_{10}k(LN{)}^{2}s}\right)}^{1/3}.$$

To evaluate the relative utility of these estimators of *ϕ*, we compared their predictions with estimates of *ϕ* obtained by computer simulations and using Equation (B1) to obtain the value of $N_{e}$ relative to *N* that best fits the observed genotypic mean. Despite the numerous assumptions in the preceding derivations, for large $L=10^{4}$ and β=0.10 to 0.60, Equation (E6a) yields estimates of *ϕ* that are generally within a factor of two of estimates derived by computer simulations if *k* is set to 1/32, and the simplified solution given by (E7b) is nearly as good (Supplemental Fig. 1).
