# Supplementary material for: The divergence of mean phenotypes under persistent Gaussian selection
Source: Genetics. 2025 Feb 25;229(4):iyaf031. doi: 10.1093/genetics/iyaf031 (PMC12005259; doi:10.1093/genetics/iyaf031)

**Supplemental Figure 1.** Comparisons of $\phi$ obtained by the semi-analytical approximation given by the solution to Equation E6a using Equation E3a, with estimates of $\phi$ determined by computer simulations and evaluation of Equation B1 to determine the values of $N_e$ that fit the data. Comparisons are only made for $Ns < L/20$, the approximate limit beyond which the system behaves in an effectively deterministic fashion.

**Supplemental Figure 2.** Estimates of $\phi = N_e/N$ obtained from the application of computer-simulation data to Equation 12, as a function of $Ns$, $L$, $\beta$, and $s$ for the case in which the optimum $\theta_S = L$ is at the extreme value of $L$ (half-Gaussian fitness function).

**Supplemental Figure 3.** Average absolute deviation of phenotypic means from the optimum, $\overline{|\delta|}$, for the case in which the optimum is $\theta_S = 3L/4$. Results from computer simulations are given for the case of no mutational bias ($\beta = 1$, open points) and $\beta = 0.33$ (closed points). Solid lower lines, given for reference, are the theoretical results for an intermediate optimum with no mutational bias (from Figure 1 in the main text). Dashed lines (barely visible, owing to the good fit to the data) are the expectations obtained by using Equation E6a with $k = (1/2)^{1/\beta}$ to obtain $\phi = N_e/N$, followed by the solution of Equations 13a,b and D2, and then Equations 15 and 8 to incorporate the additional load associated with segregating deleterious mutations. Results are shown for $s = 0.01$ and 0.00001, in both cases with $\beta = 0.33$. Note that the high plateau of the mean deviation at large $Ns$ when $L = 10$ is a consequence of the optimum being $L = 7.5$, an unobtainable value owing to the integer nature of the effects of individual mutations in the simulations.

# Supplemental Figure 1

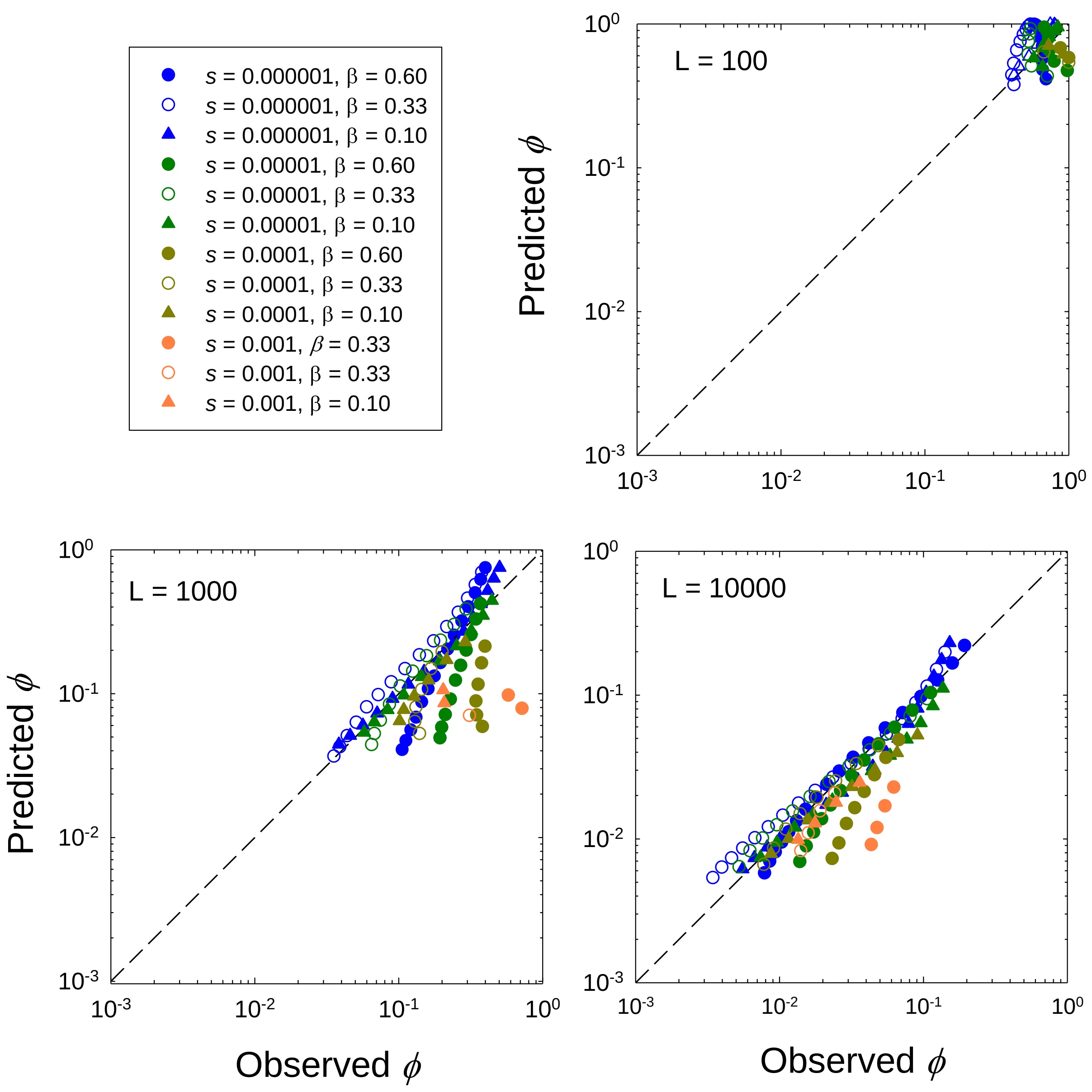

# Supplemental Figure 2

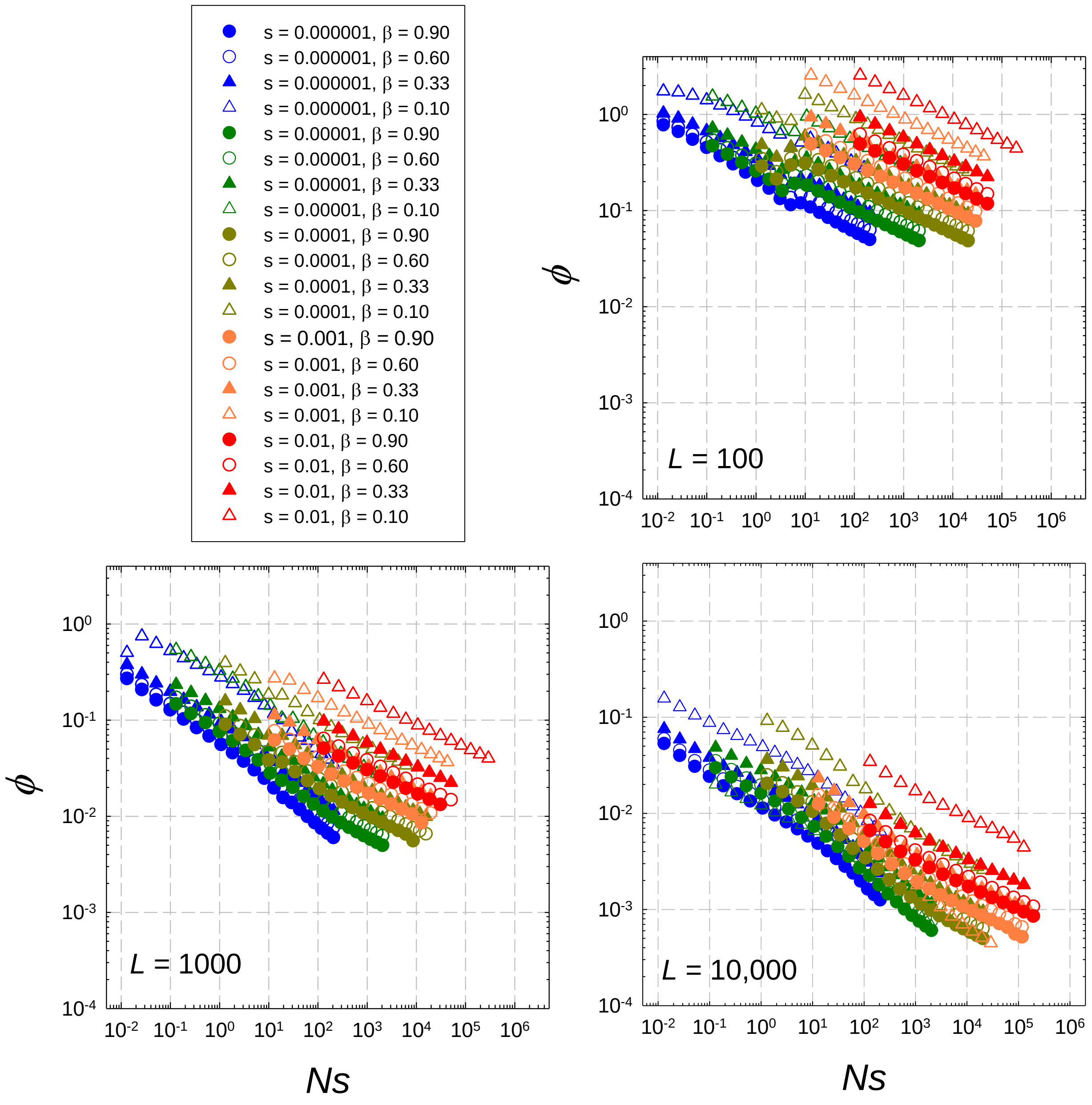

# Supplemental Figure 3

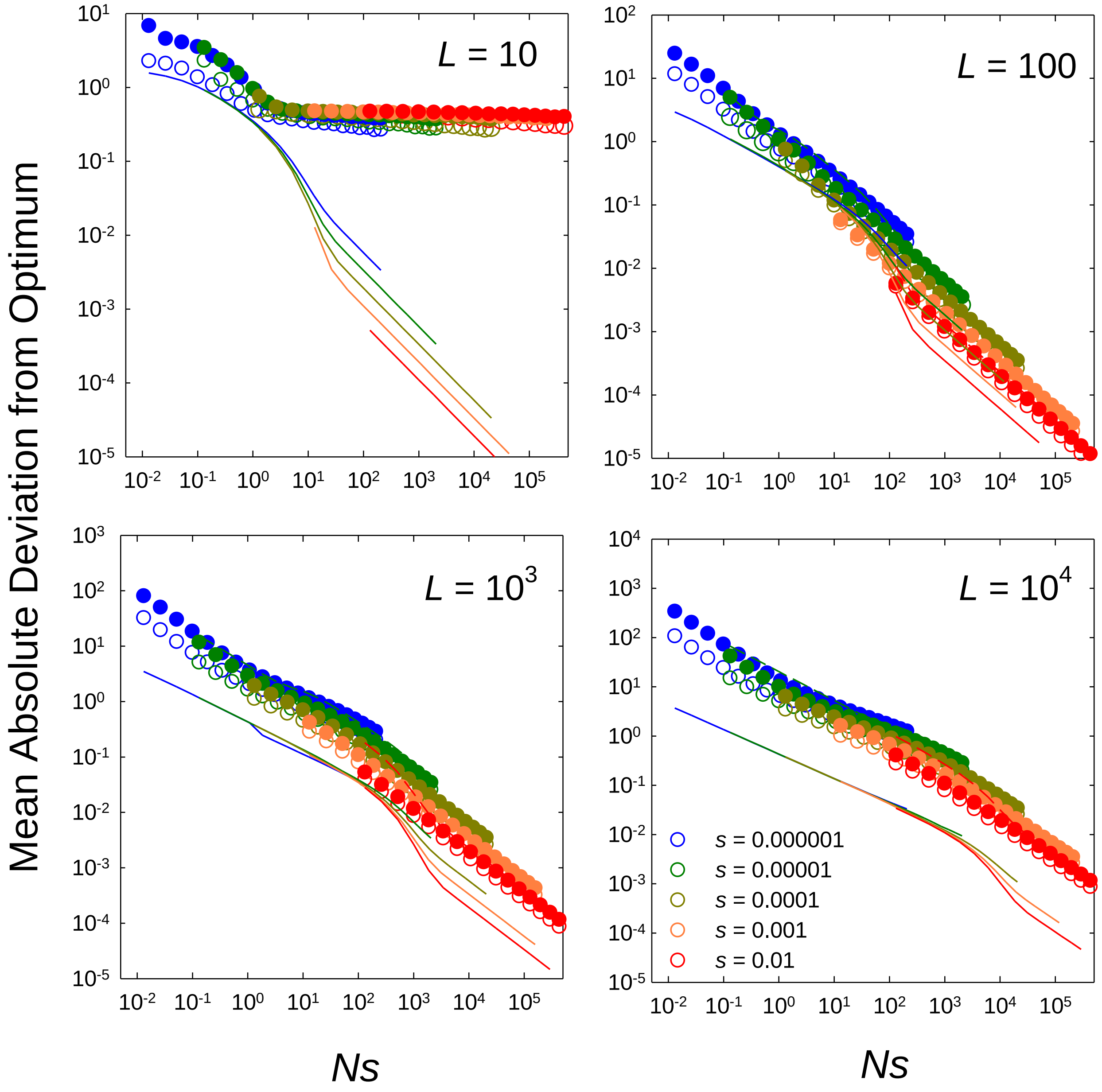

Supplement: iyaf031_Supplementary_Data [file iyaf031_supplementary_data.pdf]
